# Switching From Immediate-Release to Fractionated Dual-Release Hydrocortisone May Improve Metabolic Control and QoL in Selected Primary Adrenal Insufficiency Patients

**DOI:** 10.3389/fendo.2020.610904

**Published:** 2021-02-01

**Authors:** Francesca Delle Cese, Andrea Corsello, Marco Cintoni, Pietro Locantore, Alfredo Pontecorvi, Salvatore Maria Corsello, Rosa Maria Paragliola

**Affiliations:** ^1^Endocrinology, Università Cattolica del Sacro Cuore-Fondazione Policlinico “Gemelli” IRCCS, Rome, Italy; ^2^Scuola di Specializzazione in Scienza dell’Alimentazione, Università di Roma Tor Vergata, Rome, Italy

**Keywords:** primary adrenal insufficiency, immediate-release hydrocortisone, modified-release hydrocortisone, dual-release hydrocortisone, adrenocorticotropic hormone

## Abstract

**Objective:**

The use of once-daily dual-release HC (DR-HC) in primary adrenal insufficiency (PAI) is often associated with benefits in metabolic parameters when compared to immediate-release HC (IR-HC). In this study, we evaluated the effects on clinical, biochemical and metabolic parameters of switching from IR-HC to lower-dose DR-HC given both in once and fractionated daily doses.

**Methods:**

Twenty autoimmune-PAI subjects were included. Patients on 30 mg/day divided in three doses IR-HC regimen (group A) were switched to DR-HC 25 mg/day given in two daily doses (20 mg in the morning and 5 mg at 2.00 p.m.); patients on 25 mg/day divided in two doses IR-HC regimen (group B) were switched to DR-HC 20 mg once daily. Biochemical and metabolic parameters, BMI and quality of life (QoL) were evaluated at the baseline and six months after the switch.

**Results:**

Our small non-randomized study with short follow up showed significant benefits in both group A and group B without any apparent side-effects. After the switch to DR-HC, a significant decrease in adrenocorticotropic hormone (ACTH), HbA1c, total cholesterol, triglycerides, LDL, cholesterol, BMI as well as a significant improvement in QoL, were observed in both groups. At 6 months, ACTH levels were lower in group A while HbA1C and total cholesterol were lower in group B.

**Conclusion:**

The DR-HC is a valid and effective therapeutic strategy to improve the metabolic control and the QoL in PAI. The reduction of ACTH levels with DR-HC regimens reflects a better biochemical control of PAI, obtained by using a lower dose and more physiological HC formulation. Both once-daily and fractionated daily doses of DR-HC showed advantages compared with IR-HC formulation.

## Introduction

Primary adrenal insufficiency (PAI) represents a rare but potentially life-threatening disorder due to chronic glucocorticoid and mineralocorticoid deficiency. The autoimmune etiology of PAI is the most common form among the Caucasian population. In fact, the current estimated prevalence in Western Countries is about 100–140 cases per million (1).

Since cortisol and aldosterone are essential regulators of water and electrolyte homeostasis, PAI requires a prompt and adequate replacement therapy to avoid severe complications. However, despite optimal conventional replacement therapy, patients affected by PAI suffer from poor quality of life (QoL), comorbidities and increased mortality (2, 3). These poor outcomes are partially related to the risk of adrenal crisis, which represents the most severe complication in PAI, and even more to the increased risk of infections and metabolic diseases associated with higher daily glucocorticoid replacement doses (4).

While mineralocorticoid replacement therapy is based only on the use of the 9-alpha- fludrocortisone, several synthetic glucocorticoid formulations are available for the treatment of cortisol deficiency (5). The most crucial dilemma is to find a reasonable balance to avoid both the risk of a “hypoadrenal state” and the metabolic consequences of an overtreatment. The “ideal” replacement therapy should be based on a drug able to mimic the endogenous cortisol activity. Hydrocortisone (HC) is the most commonly used drug for PAI. Endocrine Society guidelines suggest to use HC (15–25 mg/day) or cortisone acetate (20–35 mg/day) divided into two or three daily doses: the largest dose should be given at waking time, the next dose in the early afternoon or at lunch-time and the third dose 6 h before bed-time (5). However, the pharmaco-kinetics of immediate-release HC (IR-HC) given in two or three doses a day, cannot entirely replicate the normal serum cortisol profile (6). Furthermore, the persistence of symptoms of hypoadrenalism in some patients suggests the use of higher IR-HC dose regimens than those suggested by guidelines.

In 2012, a dual-release formulation of HC (DR-HC, Plenadren ^®^) was developed to mimic the physiological cortisol rhythm more closely. This new preparation consists of an immediate-release coating (IR) and an extended-release core (ER). These two characteristics provide a physiological morning peak of cortisol concentration within 20 min after fasting intake and a smooth serum cortisol profile over the day (7). Several studies have reported some benefits for patients with PAI using DR-HC preparations in a single morning dose instead of standard IR-HC (8). However, to our best knowledge, there are no studies that have demonstrated a reduction in adrenocorticotropic hormone (ACTH) and body mass index (BMI), or an improvement in glucose tolerance, lipid profile and QoL scores in patients receiving the new formulation of HC (DR-HC) in fractionated doses. From these considerations, it could be interesting to evaluate the metabolic changes in patients switching from conventional IR-HC treatment to DR-HC in fractionated doses. Therefore, the aim of this study was to evaluate the effects on anthropometric, metabolic and hormonal parameters as well as the QoL in two groups of patients with PAI, previously treated with high IR-HC doses (25–30 mg/day) and then switched to lower DR-HC regimens (20–25 mg/day), both in single and fractionated doses.

## Materials and Methods

This was a prospective, two-armed, interventional study with a 6 months observational period. We included patients aged between 25 and 70 years with autoimmune PAI diagnosed by at least 5 years, confirmed by the presence of adrenal cortex antibodies (ACA) on stable HC treatment.

We excluded patients affected by cancer, cardiovascular disease, type 1 and type 2 diabetes and patients who were taking doses of IR-HC lower than 25 mg/day or higher than 30 mg/day or who were taking different glucocorticoid replacement therapy. We also excluded patients treated with lipid-lowering drugs or drugs interfering with HC metabolism.

Patients gave their written informed consent to participate in the study.

We enrolled 20 patients with autoimmune PAI, 14 women and 6 men - F:M = 2.3:1; median age 51.5 years (43.0–56.0; range: 32–70 years) who were followed between September 2018 and March 2019 in the Division of Endocrinology and Diabetology, Catholic University School of Medicine, Rome.

Six patients presented with isolated PAI. Thirteen patients had PAI associated with autoimmune thyroid disease (ATD). In particular, 11 out of 13 patients were affected by Hashimoto’s thyroiditis (HT) and two of 13 by Graves’ disease (GD) treated with radioiodine. All patients with ATD were on thyroxine treatment and did not need any dose adjustment because thyroid stimulating hormone (TSH) was normal before and during the study. Two patients presented PAI associated with premature ovarian failure (POF) and they were not on hormone replacement therapy (**Table 1**).

**Table 1:** Characteristics of study population.

**Case n°**	**Sex**	**Age (yrs)**	**Autoimmune thyroid diseases**	**Other autoimmune diseases**
**1**	F	52	Chronic autoimmune thyroiditis	
**2**	M	47		
**3**	M	67	Chronic autoimmune thyroiditis	
**4**	F	67	Chronic autoimmune thyroiditis	Chronic atrophic gastritis
**5**	F	70	Chronic autoimmune thyroiditis	
**6**	F	67		
**7**	M	53	Graves’ disease (treated with radioactive iodine (RAI) therapy)	
**8**	F	36		
**9**	F	46	Chronic autoimmune thyroiditis	
**10**	F	49	Chronic autoimmune thyroiditis	Premature ovarian failure (POF)
**11**	F	70	Chronic autoimmune thyroiditis	
**12**	F	57	Graves’ disease (treated with radioactive iodine (RAI) therapy)	
**13**	F	32	Chronic autoimmune thyroiditis	
**14**	F	35		
**15**	F	50	Chronic autoimmune thyroiditis	
**16**	M	39	Chronic autoimmune thyroiditis	Chronic atrophic gastritis
**17**	F	53		Premature ovarian failure (POF)
**18**	F	51	Chronic autoimmune thyroiditis	
**19**	M	65		
**20**	M	40		

All patients were on fludrocortisone 0.1 mg once daily at baseline and for follow-up period.

The 20 patients were divided in two groups according to their baseline glucocorticoid replacement regimen. Group A included patients who were on conventional thrice-daily (TID) IR-HC replacement therapy (N = 10; total daily dose of 30 mg: 15 mg at waking time, 10 mg at 2.00 p.m. and 5 mg at 4.00 p.m.) while group B included patients who were on conventional twice-daily (BID) replacement therapy (N = 10; total daily dose of 25 mg: 15 mg at waking time, 10 mg at 2 p.m.). This particular thrice daily IR-HC regimen, different to common clinical practice and medical literature, had been tailored to our patients’ needs during the past years of follow-up. Patients were on stable therapy with the above-mentioned regimens for at least 1 year before their enrollment in the study. The use of glucocorticoid replacement regimens higher (25–30 mg/day) than those proposed by the guidelines (15–25 mg/day) has been motivated by the lack of adequate clinical and laboratory control with lower IR-HC doses in the patients’ past medical history.

Patients were switched from IR-HC to lower-dose DR-HC regimens. Group A changed replacement therapy with DR-HC 25 mg/day given in two daily doses: 20 mg at waking time and 5 mg at 2.00 p.m. Group B changed replacement therapy with DR-HC 20 mg/day given once daily at waking time. The dose of DR-HC was maintained unchanged in all patients for the whole period of observation.

Clinical (BMI and QoL) and biochemical parameters [glycated hemoglobin (HbA1c), total cholesterol, high-density lipoprotein (HDL) cholesterol, triglycerides, sodium (Na), potassium (K), ACTH] were evaluated at baseline (before the switch to DR-HC) and after 6 months. Laboratory evaluation was performed in the morning, fasting, before taking any replacement therapy.

We used a single laboratory (Fondazione Policlinico Universitario Agostino Gemelli IRCCS, in Rome) to perform the biochemical analysis of all the analytes. All blood samples were analyzed in the same day of collection. ACTH was measured with Liaison^®^ Analyzer (DiaSorin, Saluggia, Italia) assay using chemiluminescent immunoassay (CLIA) technology (intra-assay and inter-assay coefficients of variation <10%). We used indirect ion-selective electrode (ISE) methods (Roche/Hitachi Cobas 8000 ISE) to investigate sodium and potassium levels. Intra-assay and inter-assay coefficients of variation (CV) for serum sodium were both <1.0% and for serum potassium were 0.34% and 1.6%, respectively.

Total cholesterol and HDL cholesterol were measured by the CHOD-PAP method (Giesse Diagnostics, Roma, Italia). For triglyceride determination GPO-PAP method (Giesse Diagnostics, Roma, Italia) was used. LDL levels were calculated using the Friedewald formula [Total cholesterol − (HDL cholesterol + (triglycerides/5)].

Blood glucose was measured using UV-Hexokinase method.

**Figure 1:**
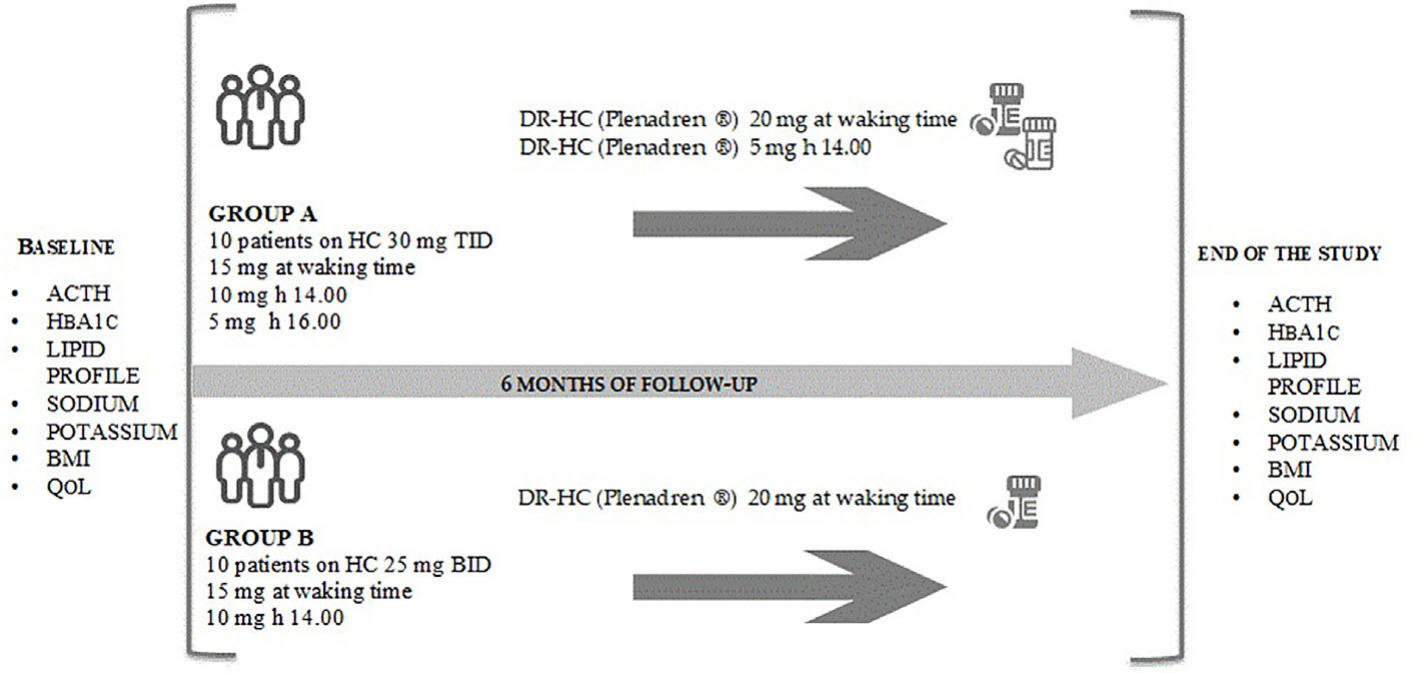
The figure reports the study design. The study is a prospective, two-armed, interventional study with a 6 months observational period. Biochemical and metabolic parameters and AddiQoL-30 test have been collected at baseline and six months after the switch to DR-HC in group A and group B. ACTH, adrenocorticotropic hormone; BID, divided in two doses; BMI, body mass index; DR-HC, dual-release hydrocortisone; HbA1c, glycated hemoglobin; IR-HC, immediate-release hydrocortisone; QoL, quality of life; TID, divided in three doses.

The reference method for HbA1c assay was high-performance liquid chromatography–mass spectrometry/capillary electrophoresis (HPLC-MS/CE), with CV of less than 2%. QoL was evaluated using AddiQoL-30 test comprising 30 items with six answers with a score ranging between 30 (lowest possible HRQoL level) and 120 (highest possible HRQoL level) (9) (**Figure 1**).

Each patient received detailed instructions for the management of emergency situations. Patients treated with DR-HC were advised to add a rescue dose of HC in case of an intercurrent illness or stress (5). Patients were educated to contact clinicians in case of severe health impairment or after glucocorticoid (GC) self-injection and to record signs or symptoms of hypoadrenal state and eventual oral HC dose adjustments on a pad that was checked at the end of the follow-up period.

### Statistical Analysis

Given the small sample size, for inferential statistical analysis, only non-parametric tests were used. Continuous variables are reported as median (25^th^ and 75^th^ percentile) while discrete variables as number (percentage). To assess differences between variables, a Wilcoxon Matched-Pairs Signed-Rank Test or a Mann-Whitney U Test were used, for paired and unpaired, respectively.

A *p* value < 0.05 was considered statistically significant.

All statistical analyses were carried out using Stata^®^ (Version 14.0, Stata Corporation; College Station, TX, USA).

## Results

DR-HC given once or twice daily, reduced ACTH levels in both group A and group B at the end of observation period [Group A: ACTH (IR-HC): 430 pg/ml (341–480) vs ACTH (DR-HC): 171 pg/ml (163–178), *p* = 0.005; Group B: ACTH (IR-HC): 850 pg/ml (662–921) vs ACTH (DR-HC): 611 pg/ml (486–686), *p* = 0.005] (**Table 2**).

**Table 2:** Plasma adrenocorticotropic hormone (ACTH) concentrations in Group A and Group B after switching from conventional hydrocortisone treatment (IR-HC) to dual-release hydrocortisone (DR-HC) in fractionated doses.

	GROUP A			GROUP B		P-value (DR-HC Group A vs Group B)
	ACTH IR-HC (pg/ml)	ACTH DR-HC(pg/ml)		ACTH IR-HC (pg/ml)	ACTH DR-HC (pg/ml)	
**Patient 1**	445	274	**Patient 1**	931	830	
**Patient 2**	480	137	**Patient 2**	831	627	
**Patient 3**	310	163	**Patient 3**	539	476	
**Patient 4**	265	120	**Patient 4**	1,058	810	
**Patient 5**	379	174	**Patient 5**	990	686	
**Patient 6**	445	263	**Patient 6**	870	469	
**Patient 7**	341	172	**Patient 7**	638	486	
**Patient 8**	415	169	**Patient 8**	860	678	
**Patient 9**	492	178	**Patient 9**	745	580	
**Patient 10**	530	168	**Patient 10**	662	594	
**Median**	430 (333–483)	171 (163–178)	**Median**	845 (656–945)	611 (486–686)	0.0002

At 6 months of follow-up after switching from conventional hydrocortisone treatment (IR-HC) to dual-release hydrocortisone (DR-HC) we compared ACTH levels in the two groups. Group A patients showed significant lower levels of ACTH compared to Group B patients [Group A vs Group B, ACTH: 171 pg/ml (163–178) vs 611 pg/ml (486–686), p = 0.0002.

We observed a significant decrease in HbA1c, total cholesterol, triglycerides, LDL, cholesterol, BMI, at the end of the 6 months treatment period with DR-HC in both groups (**Table 3**). No significant changes in HDL, cholesterol, Na and K levels were observed during DR-HC treatment. No side effects or intercurrent disease occurred during follow-up and dose adjustment was not necessary in this period.

**Table 3:** Glycated hemoglobin (HbA1c), body mass index (BMI), and serum lipids at baseline (on IR-HC treatment) and at 6 months after switching to DR-HC treatment in Group A and Group B.

**GROUP A**						
		IR-HC (tid: 15 + 10 + 5 mg)		DR-HC (bid: 20 + 5 mg)		P-value
**BMI**		24.5 (24.0–30.0)		23.5 (20.0–28.0)		0.004
**HbA1c**		41 (40–42)		37 (35–40)		0.005
**Total Cholesterol**		238 (222–245)		209 (192–220)		0.005
**HDL**		75 (68–84)		72.5 (67.0–78.0)		0.79
**Triglycerides**		134 (110–162)		100 (83–143)		0.007
**LDL**		133.7 (125.6–140.6)		115.4 (89.2–125)		0.03
GROUP B						
** **		**IR-HC (bid: 15 + 10 mg)**		**DR-HC (20 mg die)**		**P-value**
**BMI**		29 (24–33)		27 (24–32)		0.007
**HbA1c**		34 (33–38)		32 (31–35)		0.004
**Total Cholesterol**		223 (205–243)		183 (171–207)		0.005
**HDL**		50 (41–55)		60 (54–82)		0.07
**Triglycerides**		113 (102–145)		100 (87–110)		0.007
**LDL**		144 (121–177)		90 (82–113)		0.005

Data analyzed using the Wilcoxon Rank-Sum Test. Bid, divided in two doses; DR-HC, dual release HC; HDL, high-density lipoprotein; IR-HC, immediate-release hydrocortisone; LDL, low-density lipoprotein; tid, divided in three doses.

In line with current literature, we observed a statistically significant improvement in QoL evaluated by administering the AddiQoL-30 questionnaire in both group A and group B after switching to DR-HC (**Table 4**).

**Table 4:** AddiQoL-30 in Group A and B at baseline and 6 months after switching from conventional hydrocortisone treatment (IR-HC) to dual-release hydrocortisone (DR-HC).

	GROUP A		P-value (Group A DR-HC vs IR-HC)		GROUP B		P-value (Group B DR-HC vs IR-HC)
	QoL IR-HC(pg/ml)	QoL DR-HC(pg/ml)			QoL IR-HC(pg/ml)	QoL DR-HC(pg/ml)	
**Patient 1**	88	96		**Patient 1**	94	99	
**Patient 2**	85	98		**Patient 2**	99	95	
**Patient 3**	92	107		**Patient 3**	88	94	
**Patient 4**	96	110		**Patient 4**	96	100	
**Patient 5**	92	102		**Patient 5**	90	94	
**Patient 6**	96	108		**Patient 6**	94	100	
**Patient 7**	86	95		**Patient 7**	98	104	
**Patient 8**	91	98		**Patient 8**	99	102	
**Patient 9**	89	96		**Patient 9**	82	89	
**Patient 10**	94	102		**Patient 10**	90	96	
**Median**	91 (87–94)	100 (96–107)	0.005	**Median**	94 (89–98)	98 (94–100)	0.004

At the end of 6 months we compared group A with group B for all parameters evaluated in our study.

We observed lower ACTH levels in Group A patients compared to Group B patients [Group A vs Group B, ACTH: 171 pg/ml (163–178) vs 611 pg/ml (486–686), *p* = 0.0002] (**Table 2**).

Consistent with this, HbA1c and total cholesterol were lower in Group B when compared with Group A. Indeed, Group B patients received a lower and probably more physiological dose of DR-HC (i.e. 20 mg in a single daily dose). No changes in other parameters were observed (**Table 5**).

**Table 5:** Glycated hemoglobin (HbA1C), body mass index (BMI), serum lipids and quality of life (QoL) detected by AddiQoL-30 test at 6 months follow-up in Group A and B.

	Group A	Group B	P-value
**BMI**	23.5 (20.0–28.0)	27 (24–32)	0.14
**HbA1C**	37 (35–40)	32 (31–35)	0.02
**Total cholesterol**	209 (192–220)	183 (171–207)	0.03
**HDL**	72.5 (67.0–78.0)	60 (54–82)	0.22
**Triglycerides**	100 (83–143)	100 (87–110)	0.63
**LDL**	115.4 (89.2–125)	90 (82–113)	0.28
**AddiQoL-30**	100 (96–107)	98 (94–100)	0.10

Data analyzed using the Wilcoxon Rank-Sum Test. HDL, high-density lipoprotein; LDL, low-density lipoprotein.

## Discussion

Our study investigated the changes in anthropometric and biochemical parameters as well as the QoL in patients with PAI switching from conventional IR-HC treatment to lower dose of DR-HC both in single and fractionated doses. Johannsson et al. (10) have already shown that DR-HC exhibited a 19.4% lower 24 h AUC in a randomized prospective crossover study performed in 64 adults with PAI with favorable metabolic outcomes. Furthermore, we aimed to understand if switching from IR-HC to an intentional reduction of DR-HC daily dose and not to an equal dose, could guarantee significant improvements in metabolic profiles and QoL without hindering well-being and getting worse the control of PAI.

The results showed that both once-daily and fractionated daily doses of DR-HC present advantages in terms of metabolic profile and ACTH levels compared with IR-HC formulation. Furthermore, no signs or symptoms of hypoadrenalism have been reported and QoL improved in spite of total daily HC dose reduction after the switch to DR-HC formulation. In our opinion, these findings are important contributions in the complex and widely discussed topic related to the replacement therapy in hypoadrenalism. These results may open a new scenario: the possibility to shift to lower DR-HC single or fractionated dose in selected patient with favorable outcomes.

Despite recent advances in early diagnosis and treatment, Addison’s disease still represents a potentially lethal condition, with high rates of morbidity and mortality, mainly for acute adrenal failure, infection, and sudden death in patients diagnosed at a young age (11). The accurate reproduction of the endogenous cortisol rhythm is very difficult to obtain and the search for an “ideal” replacement protocol for hypoadrenal patients is still ongoing. Furthermore, objective biochemical parameters for the monitoring of the replacement therapy are lacking. Guidelines recommend only to use indirect criteria (body weight, postural blood pressure, signs of glucocorticoid excess) and blood electrolytes, to monitor replacement therapy (5). The hyperpigmentation is often associated with high ACTH levels and indicates insufficient replacement therapy (5) and ACTH should not be routinely used, but only when under- or overtreatment is suspected. In fact, the goal should not be to achieve “normal” ACTH levels, as this is known to often lead to overtreatment. Replacement therapy should seek the aim of improving QoL of hypoadrenal patients, avoiding both adrenal crisis and overtreatment, the latter being associated with worsening of metabolic profile and consequent increased cardiovascular risk. Furthermore, it has been largely demonstrated that the loss of cortisol rhythmicity per se is associated with metabolic alterations as well as with fatigue and depression (12).

In recent years many studies have reported the implications of supra-physiological doses of GC replacement therapy: low QoL (1), increased risk of osteoporosis (13) and mortality rate (11) or increased risk of cardiovascular diseases (14) or infections (15). To date, patients with PAI receive more antihypertensive and lipid lowering drugs than general population, suggesting that PAI could be an independent risk factor of cardiovascular disease (14, 16).

Once-daily DR-HC provides a cortisol exposure-time profile close to the physiological one. DR-HC can be administered once daily both at a dose of 20 mg and at higher doses. Many studies found benefits in giving DR-HC in patients with adrenal insufficiency compared to IR-HC. In particular, weight reduction and improvements in glucose profile and blood pressure have been reported (10). Recently Isidori et al., showed a weight and HbA1c reduction and an improvement in QoL and infection rates in the group switched to the same dose of DR-HC after 24 weeks of follow-up period, providing a link between innate immunity, clock genes and physiological cortisol rhythm. IR-HC therapy is associated to a reduced T-killer function that could explain morbidity and mortality from infections. On the contrary, the switch to DR-HC formulation increased CD161^+^ natural killer cell count with amelioration of chronic pro-inflammatory profile (17). Giordano et al. recently found a waist circumference, HbA1c and LDL cholesterol reduction after 12 months switch to DR-HC formulation, as well as a reduction of ACTH levels after 6 months (18). However, most studies have been performed considering once daily DR-HC dosage, while data about the use of fractionated DR-HC dosage are lacking.

In our clinical practice, DR-HC fractionated dose (20 mg at waking time and 5 mg at 2.00 p.m) can be useful in patients who are not well replaced by using a single daily dose and who complain about symptoms of hypoadrenalism in the second half of the day instead of increasing DR-HC posology or switching to IR-HC. In this study, we proposed the use of fractionated doses (20 mg + 5 mg/day in two doses) in patients who were on therapy with IR-HC 30 mg/day divided in three doses. We found a significant reduction in BMI and HbA1c as well as an improvement in lipid profile and QoL score after 6 months of treatment with DR-HC compared to baseline. These findings can be explained by the reduction in total HC administered after the switch to DR-HC. The statistically significant decrease of HbA1c and total cholesterol in Group B (DR-HC 20 mg/once daily) compared to group A can be explained by the lower dose of DR-HC used in Group B and especially by the lower cortisol exposition during the evening in once daily DR-HC regimen. Furthermore, in accordance with other studies (18, 19) that used AddiQoL-30 as an evaluation questionnaire for QoL, we documented an improvement in the QoL in both groups treated with DR-HC, although the group of patients taken into consideration is small.

Interestingly, ACTH levels did not increase, but on the contrary decreased in spite of the reduction in total daily HC dose. Before obtaining our results, we could speculate that the shift to DR-HC, which avoids supra-physiological cortisol levels, would result in a weaker negative feedback on the hypothalamus-pituitary-adrenal (HPA) axis causing consequent stable/increased ACTH levels. On the contrary, our data showed a reduction in morning ACTH levels in all patients after shifting to DR-HC. A possible explanation could be found in the DR-HC pharmacokinetics (8) which guarantees the absence of cortisol peaks, reducing the overall daily exposure to HC, reproducing more closely the physiological cortisol rhythm. Interestingly, these changes occurred both in the group of patients receiving the once daily DR-HC schedule and in the one receiving fractionated doses. Moreover, our results have also clearly documented a greater reduction of ACTH levels in Group A than Group B. This can be easily explained by the effect of the 5 mg DR-HC afternoon dose, which strengthens the pharmacodynamics effect on HPA axis in the evening, when ACTH secretion is more sensitive to glucocorticoid inhibition.

As previously mentioned, the biochemical evaluation of adequate glucocorticoid replacement therapy is still an unsolved problem. The role of ACTH in establishing adequate replacement therapy in patients with PAI is limited and literature suggests to consider it for dose adjustments only if suppressed or highly elevated (at least more than eight-nine-fold above the upper limit of the reference range) (20, 21). Urinary free cortisol (UFC), plasma cortisol, salivary cortisol and, recently, hair cortisol have poor utility in terms of tailoring cortisol replacement therapy (22–24).

Comparing patients treated with IR-HC 20 mg with a different group of patients treated with the corresponding replacement dose of cortisone acetate (25 mg), HC is associated with lower mean ACTH levels (20). An Italian study including 34 patients with PAI on cortisone acetate replacement therapy showed that ACTH was lower in patients on three daily doses than in patients on two daily doses, evidence not confirmed in other studies (25). Undoubtedly, the difficulty in the interpretation of these results should consider the different inter-individual sensitivity to glucocorticoid action, which can represent a limitation if two different groups are compared.

Despite the decrease in the daily replacement dose after the switch to DR-HC could be a bias in interpreting the results, our work suggests that in real-life clinical practice, a lower GC daily exposure without the risk adrenal crisis and signs or symptoms of adrenal insufficiency, could be obtained by switching to DR-HC in single or fractionated regimens. These findings overall reinforce the hypothesis that the metabolic advantages and the ACTH levels improvement could be related to the favorable pharmacokinetic profile of DR-HC rather than to the reduction of daily HC doses, being the condition of “under-replacement” characterized by increased ACTH levels.

Our study has some limitations: 1) the small sample size (due to the rarity of disease and the stringent exclusion criteria); 2) study design (non-randomized study); 3) the lack of a body composition assessment (with DEXA or bioelectrical impedance), considering that the decrease of BMI is not a specific parameter to evaluate variations in body composition; 4) lifestyle, dietary habits and physical activity during the follow-up period were not verified; 5) reflection of seasonal differences between autumn and spring on anthropometric, metabolic, ACTH and QoL data were not considered, representing a possible confounding factor.

However, our work has also some strengths: 1) to our knowledge this is the first study protocol in which fractionated doses of DR-HC are used in PAI; 2) the two groups of patients chosen are homogenous in terms of replacement therapies (GC and MC regimens).

## Conclusions

The DR-HC is a valid and effective therapeutic strategy to improve the metabolic control and the QoL in patients with PAI. Furthermore, another advantage is related to the reduction of ACTH levels, which reflects a better biochemical control of the disease using a lower and more physiological HC formulation. In our study both once-daily regimen therapy and fractionated daily doses of DR-HC have been investigated and both regimens showed advantages when compared with IR-HC formulation. In particular, DR-HC administered in two daily doses (20 mg in the morning and 5 mg at 2 p.m.) ensured a better control of ACTH levels, in spite of a slightly worse metabolic profile, when compared with DR-HC once-daily. However, fractionated doses regimen can be helpful in patients who require higher HC and who complain about symptoms of hypoadrenalism in the afternoon, especially if associated with hyperpigmentation and poorly-controlled ACTH levels. To reinforce these findings a randomized double-blind study should be performed in a larger group of patients.

## Data Availability Statement

The raw data supporting the conclusions of this article will be made available by the authors, without undue reservation.

## Ethics Statement

The studies involving human participants were reviewed and approved by Fondazione Policlinico Universitario Agostino Gemelli IRCCS. The patients/participants provided their written informed consent to participate in this study. Informed consent was obtained from all individual participants included in the study.

## Author Contributions

FDC, AC, PL, AP, SMC, and RMP contributed to conception and design of the study and writing of the manuscript. FDC and MC contributed to the organization of the database and performed the statistical analysis. All authors contributed to the article and approved the submitted version.

## Conflict of Interest

FDC, RMP, PL, and SMC are investigators for the EU-AIR study, which is sponsored by Shire.

The remaining authors declare that the research was conducted in the absence of any commercial or financial relationships that could be construed as a potential conflict of interest.
